# A cross sectional study of unmet need for health services amongst urban refugees and asylum seekers in Thailand in comparison with Thai population, 2019

**DOI:** 10.1186/s12939-020-01316-y

**Published:** 2020-11-11

**Authors:** Rapeepong Suphanchaimat, Pigunkaew Sinam, Mathudara Phaiyarom, Nareerut Pudpong, Sataporn Julchoo, Watinee Kunpeuk, Panithee Thammawijaya

**Affiliations:** 1 Division of Epidemiology, Department of Disease Control, Ministry of Public Health, Nonthaburi, Thailand; 2 International Health Policy Program (IHPP), Ministry of Public Health, Nonthaburi, Thailand; 3 Division of Innovation and Research, Department of Disease Control, Ministry of Public Health, Nonthaburi, Thailand

**Keywords:** Urban refugee, Asylum seeker, Unmet need, Healthcare, Thailand

## Abstract

**Background:**

Although the Thai government has introduced policies to promote the health of migrants, it is still the case that urban refugees and asylum seekers (URAS) seem to be neglected. This study aimed to explore the degree of healthcare access through the perspective of unmet need in URAS, relative to the Thai population.

**Methods:**

A cross-sectional survey, using a self-reporting questionnaire adapted from the Thai Health and Welfare Survey (HWS), was performed in late 2019, with 181 URAS completing the survey. The respondents were were randomly selected from the roster of the Bangkok Refugee Center. The data of the URAS survey were combined with data of the Thai population (*n* = 2941) from the HWS. Unmet need for health services was defined as the status of needing healthcare in the past 12 months but failing to receive it. Bivariate analysis was conducted to explore the demographic and unmet need difference between URAS and Thais. Multivariable logistic regression and mixed-effects (ME) model were performed to determine factors associated with unmet need.

**Results:**

Overall, URAS were young, less educated and living in more economically deprived households, compared with Thais. About 98% of URAS were uninsured by any of the existing health insurance schemes. The prevalence of unmet need among URAS was significantly higher than among Thais in both outpatient (OP) and inpatient (IP) services (54.1% versus 2.1 and 28.0% versus 2.1%, respectively). Being uninsured showed the strongest association with unmet need, especially for OP care. The association between insurance status and unmet need was more pronounced in the ME model, relative to multivariable logistic regression. URAS migrating from Arab nations suffered from unmet need to a greater extent, compared with those originating from non-Arab nations.

**Conclusion:**

The prevalence of unmet need in URAS was drastically high, relative to the prevalence in Thais. Factors correlated with unmet need included advanced age, lower educational achievement, and, most evidently, being uninsured. Policy makers should consider a policy option to enrol URAS in the nationwide public insurance scheme to create health security for Thai society.

**Supplementary Information:**

The online version contains supplementary material available at 10.1186/s12939-020-01316-y.

## Background

At present, cross-border mobility is a soaring global trend for many reasons, including people searching for better economic prospects, and escaping from war and political conflicts. In 2017, international cross-border populations amounted to 258 million (3.4% of global population) [1]. Of these 258 million, 68 million were forcibly displaced people. Of that 68 million, 25 million were refugees and three million were asylum seekers [2]. The situation of refugees has gained increasing attention in the global health field in recent years, particularly since the 2011 Syrian crisis which resulted in more than 6 million refugees fleeing from Syria to Europe [3]. Asia is another region that has encountered a refugee crisis. An obvious case is the exodus of more than 700,000 Rohingya refugees from Rakhine State in Myanmar to Bangladesh, during 2015–2017 [4].

The United Nations (UN) and the World Health Organization (WHO), as well as many other international development partners, have called for more concrete actions to protect refugees’ rights to health and well-being. Some tangible outputs of these actions include the launch of the World Health Assembly (WHA) Resolution 70.15, entitled ‘Promoting the health of refugees and migrants’ [5], the New York Declaration for Refugees and Migrants [6, 7] and, recently, the Global Compact on Refugees in 2018 [8].

Thailand is one of the most popular destinations for international migrants and refugees in Southeast Asia. The majority of migrants are workers from Cambodia, Lao PDR, Myanmar and Vietnam (CLMV collectively). Some of them have entered the country unlawfully and are known as undocumented migrants. It is estimated that today, there are more than three million migrant workers living in Thailand [9].

The Thai government has implemented policies to protect the well-being of undocumented migrants for several years. One remarkable policy is the One Stop Service (OSS) registration measure for undocumented CLM migrants and their dependants [10]. Migrants who register with the OSS have their profile recorded in the civil registry and acquire a work permit, alongside undertaking nationality verification (NV). The Ministry of Public Health (MOPH) also instigated a nationwide public insurance policy, called the ‘Health Insurance Card Scheme’ (HICS), for these registered migrants and their dependants. The HICS benefit is comprehensive, covering inpatient (IP) care, outpatient (OP) care, high-cost care, disease prevention and health promotion [11].

According to the National Security Act, all Thai nationals are covered by one of the three main public insurance arrangements: (i) Civil Servant Medical Benefit Scheme (CSMBS) for civil servants; (ii) Social Security Scheme (SSS) for employees in the formal sector; and (iii) the Universal Coverage Scheme (UCS) for those who are not covered by the CSMBS and the SSS. With the function of the HICS (for registered CLM migrants) and the insurance schemes for Thais (USMBS, SSS and UCS), Thailand (in principle) has achieved Universal Health Coverage (UHC) for almost everybody within its territory [12, 13].

While undocumented migrants seem to be in the spotlight of health policies in Thailand, refugees and asylum seekers are often neglected [14]. None of the aforementioned policies include refugees and asylum seekers. The situation is more complicated among refugees and asylum seekers in urban areas compared with those in temporary shelters. This is because implementing health measures in a well-defined geographical space is relatively straightforward, and local healthcare providers are well aware of the existence of refugees in the camps. Besides, the United Nations High Commissioner for Refugees (UNHCR) and a number of international non-governmental organizations (NGOs), such as Médecins Sans Frontières and the International Rescue Committee, in coordination with public facilities along the border, have provided humanitarian assistance in the refugee camps for years [15, 16].

Unlike refugees in temporary shelters, urban refugees and asylum seekers (URAS) received little attention within the public health sphere in Thailand. Almost all URAS are residing in Bangkok, under the patronage of the United Nations High Commissioner for Refugees (UNHCR). So far, there are about 5000 URAS and 97,000 refugees in temporary shelters [17, 18]. URAS are neither covered by the HICS, nor by the public insurance schemes originally designed for Thais. Nonetheless, some private facilities or insurance companies have initiated a health insurance package for URAS, which are conditional upon affordability. Some media or local NGOs suggest that URAS in Thailand face many hindrances in accessing health services, for instance, poverty, language difficulty, and precarious citizenship status [19, 20]. Moreover, some government officials are unaware of the existence of URAS [19]. Finally, there is no systematic evaluation of the degree of healthcare access for URAS in Thailand.

Therefore, the objective of this study is to explore the degree of healthcare access among URAS, in comparison with the Thai population. In this regard, we use ‘unmet need’ for health services as an indicator to gauge the ability to access health care. The concept of unmet need originates from the reproductive health field, but during the past two decades, its application has become widespread to other fields, including population health and critical care [21–23].

## Methods

### Study design, populations and samples

**Fig. 1:**
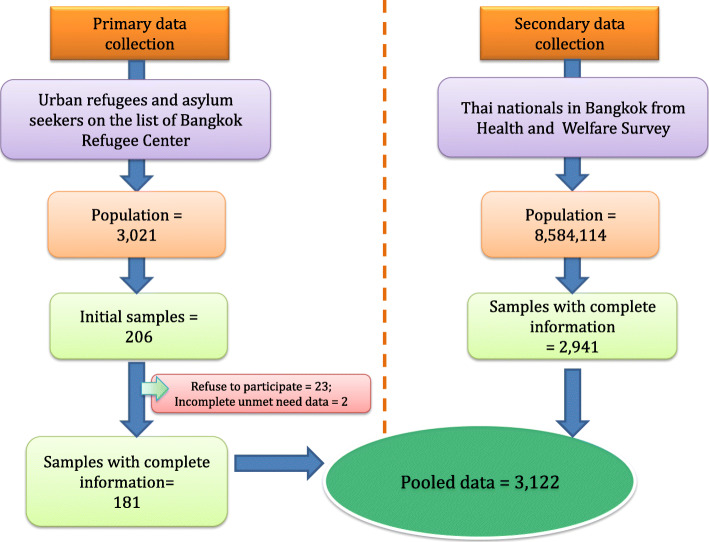
Population frames, samples and data sources

Both primary and secondary data collection was applied. We performed a cross-sectional survey on URAS from October to December 2019, and examined prior survey data on the Thai population through the 2019 Health Welfare Survey (HWS). HWS is a nationwide biennial survey jointly conducted by the National Statistical Office (NSO) and the International Health Policy Programme (IHPP) of the MOPH. We first contacted the Bangkok Refugee Centre (BRC), a charitable agency in collaboration with UNHCR, whose work is to support the well-being of URAS. For this study, we focused on URAS of the top-ten most common nationalities in Thailand: namely, Pakistani, Vietnamese, Cambodian, Somali, Afghan, Palestinian, Chinese, Sri Lankan, Iraqi, and Syrian, comprising 3021 URAS in total. We then sampled 206 URAS from the pool of 3021 URAS in the BRC roster (more details in *‘Sample size calculation, sampling methods and survey design’*). Among these 206 samples, 181 completed the survey questionnaire. Once the primary survey on URAS was completed, we combined the data of these 181 URAS with Thai data from HWS, focusing on those living in Bangkok (*n* = 2941). The final dataset comprised 3122 observations in total, Fig. 1.

### Sample size calculation, sampling methods, and survey design

We used the prevalence of unmet need for healthcare as the main indicator for sample size estimation. The following formula, $n=\frac{{\left({Z}_{1-\frac{\alpha }{2}}\sqrt{2 PQ}+{Z}_{1-\beta}\sqrt{P_1{Q}_1+{P}_2{Q}_2}\right)}^2}{{\left({P}_1-{P}_2\right)}^2}$ was used; where *α* = 0.05; *β* = 0.2 ${Z}_{1-\frac{\alpha }{2}}$ = 1.96; *Z*_1 − *β*_ = 0.84; *P*_1_ = 0.11, *Q*_1_ = 1- *P*_1_; *P*_2_ = 0.012, *Q*_2_ = 1- *P*_2_; P = (*P*_1_ + *P*_2_)/2 and Q = 1-P. *P*_1_ refers to the unmet need prevalence in URAS whereas *P*_2_ refers to similar prevalence in the Thai population. The most recent data on unmet need in Thai citizens suggested a prevalence of 1.2%, according to Thammatacharee et al. [24]. Thus *P*_2_ was replaced by 0.012. As there has been no study on unmet need among URAS in Thailand, we searched for the indicator in studies outside Thailand. We found a piece of work by Busetta et al., which examined the prevalence of unmet need of refugees in Italy while applying the same unmet need questions as the Thai HWS [25]. Busetta et al. reported that the degree of unmet need in refugees was about 11%. Hence we substituted 0.11 for *P*_1_. It should be noted that both HWS and the Italian survey followed the original questions proposed by the European Union Statistics on Income and Living Conditions (EU-SILC). Taking into account a 20% non-response rate and incomplete information, at least 140 samples were needed in each sample group (URAS and Thais).

**Fig. 2:**
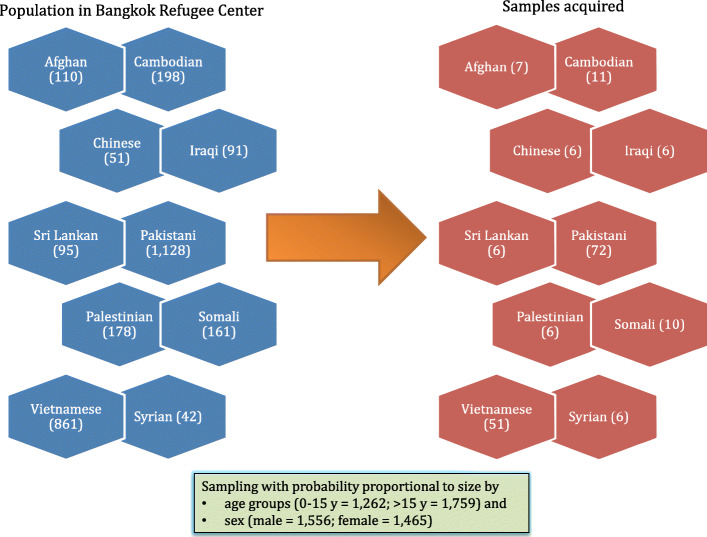
Number of samples participating in the survey sorted by nationalities

The existing records of Thai respondents in HWS already outnumbered the required number of samples; therefore no further sampling was required. For URAS, we used stratified random sampling with probability proportional to size (PPS), according to age group, sex and nationality. Fortunately, in the fieldwork, the BRC officers informed us that they were capable of recruiting 206 participants. We therefore expanded the sample size to the suggested number. However, during the survey process, 23 URAS refused to take part. Of the remaining 183, two did not complete the unmet need questions. As a result, only 181 URAS were enrolled in the study. Figure 2 displays the overview of total population in each nationality from the BRC list and the actual samples acquired. Details of the sample volume tallied by age groups, nationalities, and sex can be found in Supplementary file 1.

All selected participants were asked to travel to BRC to complete the paper questionnaire. The investigators provided financial support to cover the travelling cost of the participants (about US$ 9). For those who had difficulty travelling, a phone interview was performed instead. For a child below 15 years of age, parents or legal guardians would respond on his or her behalf. The questionnaire was translated into the respondents’ own language. For those who had difficulty reading, a verbal interview was performed in place of a written questionnaire. On average, each respondent took approximately 30 min to complete the questionnaire. A focal coordinator was prepared for each nationality group. These coordinators were volunteers working with BRC. Preparatory meeting between the research team and focal coordinators was arranged prior to the survey in order to fine-tune understanding and to assess the survey feasibility.

### Operational definitions

We set operational definitions as follows. Firstly, ‘refugee’ is a person who has been forced to flee his or her country because of persecution, war or violence, and his or her request for sanctuary is ratified by the UNHCR according to the 1951 Refugee Convention [26]. Secondly, asylum seeker means someone who has been forced to flee his or her country because of persecution, war or violence and his or her request for sanctuary has yet to be processed by the UNHCR according to the 1951 Refugee Convention [26]. Lastly, unmet need refers to a status where a person reported that he or she needed health examination or treatment for any type of health issue within the past 12 months, but he or she did not receive or did not seek it. This definition is adapted from the original unmet need survey by EU-SILC [27].

### Questionnaire and determinants of interest

The questionnaire for the URAS survey was adapted from the HWS questionnaire. Two rounds of consultative meetings between the research team, health system academics and BRC staff were arranged to ensure content validity and to make sure that the participants clearly understood the questions. The questionnaire contained two domains: (i) an individual’s demography and (ii) unmet need for health services.

Questions about an individual’s demography (1st domain) consisted of sex, age, insurance status (insured with either public or private insurance versus uninsured); education background (primary level, secondary level, and degree or above), and household monthly income. For convenience, we classified age into age groups (≤ 15 years, > 15 but ≤ 60 years, and > 60 years) and created a new binary variable, called ‘household economy’, using a cut-off at 45,707 Baht (US$ 1428) - the average monthly income of a household in Bangkok according to the NSO [28].

Questions about unmet need for health services (2nd domain) asked a respondent to self-assess if, during the last 12 months, he or she had felt unwell and needed healthcare but did not receive it. These questions were sub-divided into OP care and IP care. Then, any respondent who experienced unmet need, was asked to recount the most important reason for not acquiring healthcare. Some examples of the reasons included ‘cannot afford treatment cost’, ‘long waiting times’, ‘no time to seek treatment’, ‘too far to travel’, and ‘do not trust health staff’.

### Statistical analysis

All statistical analyses were performed by Stata v14.0 (StataCorp LP, College Station, Texas, US—serial number: 401406358220). We divided the analysis into two parts: (i) descriptive statistics and (ii) inferential analysis. In the first part, all categorical variables were expressed as frequency and percentage. Age and household income were presented by median and interquartile range (IQR).

In the second part, we commenced with bivariate analysis, using Chi-square or Fisher’s exact test (for categorical variables) and Mann-Whitney U test (for continuous variables), to identify: (a) the demographic difference between URAS and Thais; and (b) the relationship between unmet need and each demographic variable.

Further, we performed multivariable logistic regression by regressing odds of unmet need in natural logarithm scale on the selected independent variables all at once. The independent variables enrolled in this step were those exhibiting *P*-value of less than 0.2 in the former bivariate analysis. For a dummy variable with three or more scales (such as age group and education achievement), if there was at least a sub-scale variable showing *P*-value of less than 0.2 in the bivariate analysis, the variables at all scales would be included in the multivariable logistic regression.

We also conducted mixed-effects (ME) logistic regression, having done multivariable logistic regression at a prior stage. This time, the ME model took the nationalities of the participants into account. We categorised nationalities into three main clusters: Thai, non-Arab Asian, and Arab Asian.

The results were presented in terms of crude and adjusted odds ratios (OR) with 95% confidence interval (CI). Inverse probability weighting was applied when assessing statistical significance in order to take the survey design into account.

### Subgroup analysis

Subgroup analysis was exercised by limiting the analysis on URAS. We then broke down the degree of unmet need by nationalities and types of URAS (urban refugee versus asylum seeker). The analysis was performed in the same fashion as the full-sample analysis.

## Results

### Demographic profiles

In total, we enrolled 3122 records in the analysis. Of these 3122 observations, 181 (5.8%) were URAS. Amongst 181 URAS, 160 (88.4%) were refugees and 21 (11.6%) were asylum seekers. Pakistanis constituted the largest single group of all URAS (39.8%), followed by Vietnamese (28.2%) and Cambodians (6.1%). The male to female ratio appeared to be similar in both Thais and URAS. About a third of Thai respondents had received primary education (34.6%), compared with 63.5% in URAS. The median age of Thais was 42 years and almost one fifth of them fell in the elderly category. In contrast, the median age of URAS was roughly 23 years with much a smaller proportion of elderly people (6.1%). The monthly household income of Thais was, on average, five times as large as that of URAS. Almost all URAS (98.7%) had a monthly household income less than the average income of most people in Bangkok. The insurance status of Thais was also in stark contrast to that of URAS. While over 99% of Thai respondents were covered by either public or private insurance, approximately 98% of URAS were completely uninsured. Only four URAS were insured, and answered in the questionnaire form that they held voluntary insurance from a private hospital in Bangkok. All of these demographic variables, except sex, yielded a statistically significant difference. Note that the number of missing data in each variable was negligible (less than 1% of the observations), except household income which appeared to be missed in over half of the samples, Table 1.

**Table 1:** Demographic characteristics of the participants^a^

Variable	Thai (*n* = 2941)	URAS^b^ (*n* = 181)	*P*-value	Test
Sex—n (%)			0.975	Chi-square
● Female	1550 (52.7)	95 (52.5)		
● Male	1391 (47.3)	86 (47.5)		
Education—n (%)			< 0.001	Chi-square
● Up to primary	981 (34.6)	115 (63.5)		
● Up to secondary	1091 (38.5)	46 (25.4)		
● Degree or above	765 (26.9)	20 (11.1)		
Median age—years (IQR)^c^	42.0 (31.0)	23.1 (27.3)	< 0.001	Mann-Whitney U
Age group—n (%)			< 0.001	Chi-square
● ≤15 years	349 (11.9)	68 (37.6)		
● >15 but ≤ 60 years	2033 (69.1)	102 (56.3)		
● >60 years	599 (19.0)	11 (6.1)		
Median household income—Baht (IQR)	30,000 (30,000)	6000 (4500)	< 0.001	Mann-Whitney U
Household economy			< 0.001	Fisher’s exact
● Above average	271 (23.9)	2 (1.3)		
● Below average	861 (76.1)	151 (98.7)		
Insurance status			< 0.001	Fisher’s exact
● Uninsured	6 (0.2)	177 (97.8)		
● Insured	2935 (99.8)	4 (2.2)		

Note: ^a^Missing data were not included in the table; ^b^Urban refugee and asylum seekers; ^c^Interquartile range

### Unmet need profiles

**Fig. 3:**
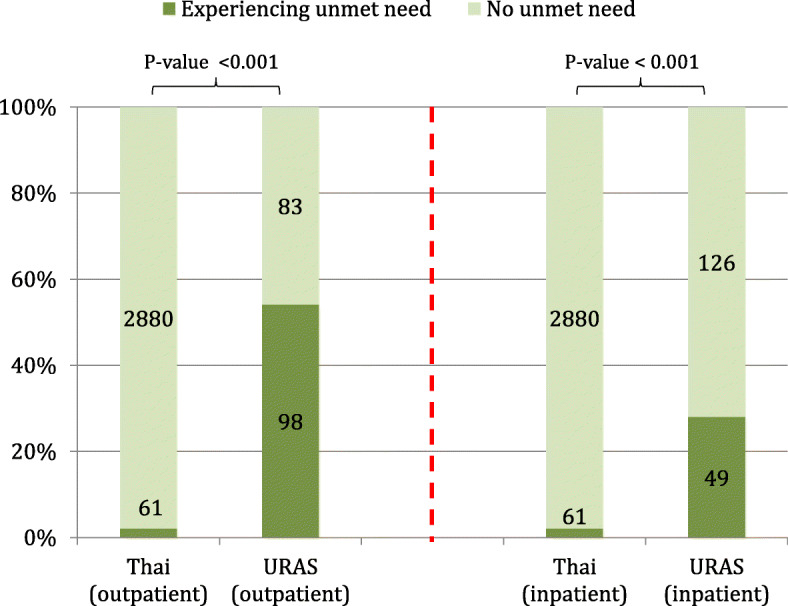
Prevalence of unmet need in Thais versus urban refugees and asylum seekers. Note: URAS = urban refugee and asylum seeker

We estimated prevalence of unmet need by dividing the number of respondents who reported that they had faced unmet need in the past 12 months by the total number of respondents. The unmet need prevalence for Thais was about 2.1% in both OP and IP health services. The unmet need prevalence for URAS in IP care was approximately 28.0%, while the corresponding prevalence in OP care was 54.1%. The difference of unmet need between URAS and Thais demonstrated strong statistical significance (*P*-value < 0.001 in both types of care), Fig. 3.

### Determinants of unmet need

The results in bivariate analysis and multivariable logistic regression were relatively similar. In OP care, being uninsured demonstrated a strong and significant association with unmet need (adjusted OR = 4.0 [95% CI = 1.5–10.6]). The odds of experiencing unmet need became lower among those with high education backgrounds. For instance, participants completing secondary education faced only half the odds of unmet need relative to those who completed only primary education (adjusted OR = 0.5 [95% CI = 0.2–0.9]). The likelihood of unmet need in the middle age group was about 2–3 times higher than for children and juveniles (crude OR = 2.7 [95% CI = 1.0–7.1;] adjusted OR = 1.9 [95% CI = 1.0–3.6]). Sex and household economy did not show a significant association with unmet need. In IP care, the findings appeared to follow the same direction as OP care. The only difference was that the relationship between insurance status and unmet need turned out to be non-significant despite maintaining a positive association (adjusted OR = 1.9 [95% CI = 0.7–5.1]).

The findings from the ME model also demonstrated a similar pattern to results from the multivariable logistic regression, though with a marginal difference in the degree of effect size. For OP care, the adjusted OR amongst the middle age group slightly declined from 2.7 (95% CI = 1.0–7.1) in multivariable logistic regression to 1.9 (95% CI = 1.0–3.6) in the ME model, while still keeping statistical significance (*P*-value = 0.049 in multivariable logistic regression and 0.041 in the ME model). The models showed almost similar results. The most noticeable difference was the adjusted OR of the insurance variable in the ME model, which expanded about three to four times, relative to the ratio in multivariable logistic regression (adjusted OR = 14.5 [95% CI = 2.6–84.1] for OP care; and adjusted OR = 10.4 [95% CI = 1.9–55.6] for IP care). Statistically significant relationship between insurance status and unmet need was observed for both types of care (*P*-value = 0.003 for OP care, and 0.006 for IP care), Tables 2, 3.

**Table 2:** Factors associated with unmet need for outpatient care

Factors	Bivariate analysis by Chi square test		Multivariable logistic regression		Mixed-effects model	
	Crude OR (95% CI)	*P*-value	Adjusted OR (95% CI)	*P*-value	Adjusted OR (95% CI)	*P*-value
Uninsured (v insured)	4.0 (1.6–9.7)	0.003	4.0 (1.5–10.6)	0.005	14.7 (2.6–84.1)	0.003
Male (v female)	1.5 (0.8–2.6)	0.163	1.5 (0.9–2.7)	0.136	1.1 (0.7–1.6)	0.770
Age group (v ≤ 15 years)						
● >15 but ≤ 60 years	2.6 (1.1–6.1)	0.031	2.7 (1.0–7.1)	0.049	1.9 (1.0–3.6)	0.041
● > 60 years	1.8 (0.6–5.1)	0.268	1.3 (0.5–3.8)	0.615	1.4 (0.7–3.1)	0.538
Education level (v primary education)						
● Secondary education	0.7 (0.3–1.3)	0.211	0.5 (0.2–0.9)	0.047	0.6 (0.3–1.0)	0.032
● Degree of above	0.4 (0.2–0.9)	0.024	0.3 (0.1–0.7)	0.005	1.0 (0.6–1.7)	0.881
Below-average economic level (v above average)	1.6 (0.7–4.0)	0.286	–	–	–	–

**Table 3:** Factors associated with unmet need for inpatient care

Factors	Bivariate analysis by Chi square test		Multivariable logistic regression		Mixed-effects model	
	Crude OR (95% CI)	*P*-value	Adjusted OR (95% CI)	*P*-value	Adjusted OR (95% CI)	*P*-value
Uninsured (v insured)	1.9 (0.8–4.7)	0.155	1.9 (0.7–5.1)	0.200	10.4 (1.9–55.6)	0.006
Male (v female)	1.5 (0.8–2.6)	0.165	1.5 (0.9–2.7)	0.144	1.0 (0.7–1.5)	0.950
Age group (v ≤ 15 years)						
● >15 but ≤ 60 years	2.6 (1.1–6.4)	0.030	2.7 (1.0–7.4)	0.047	1.4 (0.7–2.7)	0.321
● > 60 years	1.8 (0.6–5.3)	0.264	1.3 (0.5–3.9)	0.591	0.9 (0.4–2.0)	0.803
Education level (v primary education)						
● Secondary education	0.7 (0.3–1.3)	0.217	0.5 (0.2–1.0)	0.047	0.6 (0.3–1.0)	0.055
● Degree of above	0.4 (0.2–0.9)	0.025	0.3 (0.1–0.7)	0.005	0.9 (0.5–1.6)	0.766
Below-average economic level (v above average)	1.6 (0.7–3.9)	0.297	–	–	–	–

Among the 98 URAS who reported unmet need for OP care, 94 (95.9%) ascribed the inaccessibility of health services to unaffordable treatment cost. The remaining four URAS raised other reasons, such as language barriers, and a fear of being arrested by the police. Of the 61 Thais who reported unmet need for IP care, 38 (62.3%) pointed towards long waiting times as the most important cause for inaccessibility. The second most important reason was dissatisfaction with the facility’s performance (11.5%). The most important reason raised for inaccessible IP care was very close to that for OP care: ‘lack of money’ in 93.9% of URAS and ‘long waiting times’ in 62.3% of Thais.

### Subgroup analysis

**Fig. 4:**
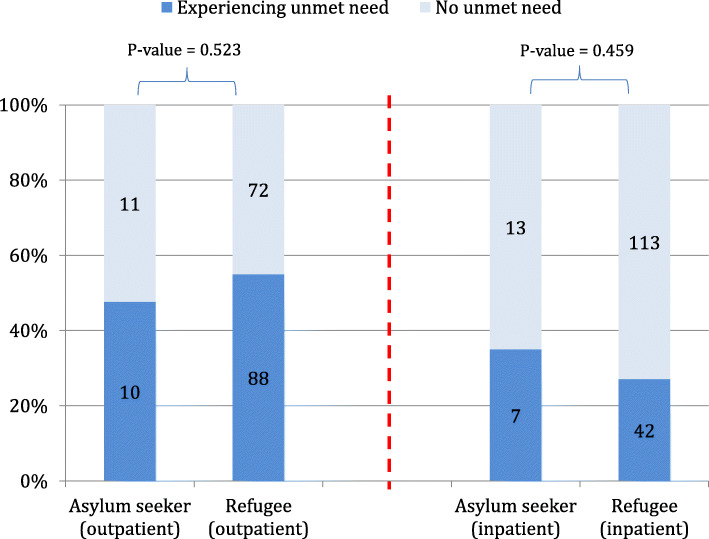
Prevalence of unmet need in urban refugees versus asylum seekers

Subgroup analysis found that, for OP care, the proportion of urban refugees facing unmet need (55.0%) was slightly larger than the corresponding proportion amongst asylum seekers (47.6%). For IP care, about one third of the participants experienced unmet need (27.1% for urban refugees and 35.0% for asylum seekers). No statistical significance difference was observed in either type of care when comparing urban refugees with asylum seekers (*P*-value = 0.523 for OP care and 0.459 for IP care), Fig. 4.

**Fig. 5:**
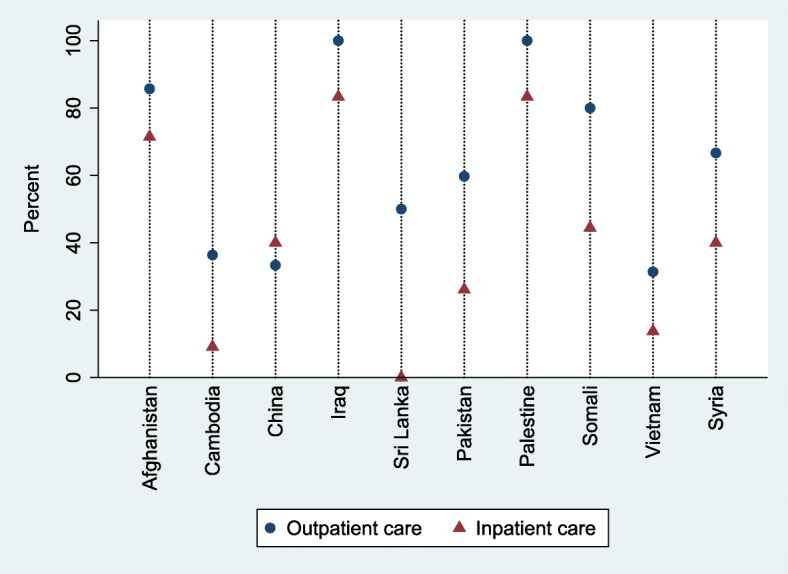
Prevalence of unmet need by nationalities

Afghans, Iraqis, and Palestinians were the populations with the greatest degree of unmet need (85.7–100.0% in OP care and 71.4–83.3% in IP care). In contrast, URAS from Cambodia and Vietnam showed the smallest unmet need estimate (31.4–33.3% in OP care and 9.1–13.7% in IP care), in relation to other nationals, Fig. 5.

## Discussion

To our knowledge, this piece of work is among the first few studies in Asia that quantitatively investigate the degree of healthcare access among URAS through the perspective of unmet need. From a macro-perspective, demographic data showed that most URAS were relatively younger, were less educated, and were living in more economically deprived households.

The evidence from this study suggests that about one fifth to one quarter of URAS faced unmet need for health services while the prevalence of unmet need in the Thai population was very small. This is not surprising; but a more interesting point is whether the degree of unmet need in URAS was larger than for other types of refugees or non-Thai populations. Unfortunately, we could not find peer-review studies on unmet need amongst any kinds of refugees in Thailand, published in the last decade. The only evidence we could identify is a study by Thein and Theptien, which reported that the prevalence for access to family planning amongst Myanmar migrant women in Bangkok was 15.8% [29]. This figure was still far lower than the prevalence found in our study (28.0% for IP care and 54.1% for OP care). Hence it is not an exaggeration to state that URAS are one of the most vulnerable groups in Thailand, even among non-Thais populations, let alone when compared with Thai citizens.

Determinants that potentially contributed to unmet need included increasing age, less education, and, most prominently, the lack of health insurance. This finding is in line with those from some other studies. Wang et al. suggested that more education was negatively associated with unmet need for supportive care among Chinese women [30]. Hailemariam and Haddis also flagged that low levels of education resulted in increasing degrees of unmet need for family planning in the Ethiopian population [31]. Bhattathiry and Ethirajan reported that unmet need for family planning decreased as age advanced [32]. This finding contradicts our discovery, which found that people with advanced age were more likely to have unmet need than those in lower age groups. Some of the explanations for this phenomenon is, first, the difference in the care of interest between our survey (focusing on IP and OP care in general) and Bhattathiry and Ethirajan’s survey (focusing only on family planning); and second, the in-house intervention of BRC.

Based on our discussion with BRC staff, we found that BRC had created its own supportive measures for URAS by allowing children up to 5 years of age to enjoy free healthcare at public facilities. Parents of these children could be reimbursed for the full healthcare cost from BRC if their children visited a health facility. This might be a reason why our findings suggest a negative association between age and unmet need. Furthermore, BRC also offered partial financial support for URAS who were admitted to a public hospital. The authority pledged to subsidise the cost of IP care for URAS up to 20,000 Baht (US$ 625) per visit. This initiative might explain why being uninsured showed significant association with unmet need for OP care, but not for IP care, in multivariable logistic regression. It is worth noting that these in-house policies have not been systematically managed as an insurance scheme and still function as charitable activities, depending on financial resources of the organisation and ad hoc negotiation with the healthcare providers.

Another interesting point from our findings was that insurance status appeared to be the most influential determinant of unmet need. The multivariable logistic regression indicated that the risk of facing unmet need for OP health services in the uninsured was about four-times as large as the risk in the insured. The degree of association became much stronger (approximately 15 times for OP care and 10 times for IP care) when applying the ME model. As, so far, there is no public insurance policy for URAS, it is not surprising that the prevalence of unmet need in URAS was staggering. This finding also corresponds with the fact that the majority of URAS pointed towards financial difficulties to afford the treatment cost as the most important concern. In other words, URAS are at huge risk of impoverishment at any time when they seek treatment, and it means that Thailand has not yet achieved UHC for everybody in its territories, as intended [33]. Since the concept of UHC covers not only the provision of essential quality health services, but also the prevention of impoverishment from healthcare spending, the issue of URAS accessing health care has considerable policy implications. Thailand is committed to the Sustainable Development Goals (SDG), including SDG target 3.8, which focuses on UHC [34]; therefore policies to enrol URAS in a public health insurance scheme should be seriously considered. In addition, leaving URAS uninsured potentially results in low access to essential healthcare, and this may undermine the health security of Thai society as a whole. Experiences from other countries that offer health insurance for URAS, such as Iran and Malaysia, are of great value and warrant further exploration [35, 36].

As Thailand is not a party to the 1951 Refugee Convention [37], the Thai government is neither obliged to guarantee any health measures for urban refugees, nor for asylum seekers whose application for refugee status is still in process. The subgroup analysis reflected this fact, showing no significant difference in the unmet need for healthcare in urban refugees, relative to asylum seekers.

Despite not being a primary objective of the study, the varying degree of unmet need among diverse national groups was thought-provoking. This was evidenced by the fact that the adjusted OR in the ME model, which had already considered the clustering effect of nationalities on unmet need, greatly expanded, compared with the ratio in the multivariable logistic regression, which assumed no correlation between observations.

The descriptive subgroup analysis also showed that Cambodian and Vietnamese URAS suffered least from unmet need, compared with other nationals. A possible explanation is that URAS from Southeast Asia nations may have lifestyle and beliefs close to Thais (including the Buddhist belief); and that Thai society is already acquainted with migrants travelling from neighbouring countries (especially from CLMV nations). In contrast, URAS from Arab nations (for instance, Iraqis, Palestinians and Syrians) presented a relatively large degree of unmet need. As Arab people are the minority in Bangkok, they possibly need a huge adaptation to incorporate the Arab way of life to South East Asian culture. This picture alludes to the concept of acculturation proposed by a great deal of prior research [38–40]. That is, refugees who can assimilate or integrate themselves into a new culture tend to have better health outcomes, compared with the poorly adjusted ones [38–40]. However, a thorough qualitative study or ethnographic research is needed to prove this presumption.

The methodology of this study bears some strengths and limitations. Regarding strengths, the study employed a systematic approach for data sampling, and we recruited participants from a household level, even though there were no physical visits to the participants’ households. Another strength of the study is the use of Thai respondent data as a comparator. We would not have a clear view on the extent of unmet need for health services in URAS had the comparator (HWS data) been missing.

However, there remain some limitations. Firstly, as the nationalities of URAS are vastly diverse, we could not guarantee a perfect translation of the questionnaire. This problem would rarely occur in the HWS questionnaire as Thai is the only formal language for Thai citizens. Nonetheless, we tried to minimize language barriers by arranging a training workshop for the survey volunteers to achieve mutual understanding between the volunteers and the research team. These volunteers mostly worked with BRC and some of them were also URAS.

Secondly, the unmet need question inquired about a history of healthcare access in the past 12 months, and therefore a recall bias was inevitable. This problem might not severely undermine the validity of the analysis as the bias could be present in both the URAS survey and the HWS. However, the bias might be more pronounced in the URAS survey compared with the HWS, because of the difference in survey practice. In our URAS survey, when we recruited people with difficulties travelling, we asked a surrogate respondent to answer the questionnaire on their behalf. In contrast, the HWS surveyors always visited the participants at their households, resulting in a lower reliance on surrogate respondents in comparison with the survey on URAS.

Thirdly, as mentioned earlier, we did not perform a physical visit to the participants’ households. This is because many URAS had precarious immigration status. Some of them were over-stayers. A physical visit meant that they needed to disclose their residential address to the surveyors. This issue was thoroughly discussed with the ethics committee and the BRC staff before the start of the fieldwork. With this limitation, some key household information that necessitates direct observation, such as household infrastructure and owner’s equity, was missing. Such information serves as the main ingredient for estimating household prosperity through the indicator called ‘asset index’ [41]. The lack of this indicator, in combination with a fair amount of missing data on household economy, might explain why the economic wealth of URAS did not exhibit a statistically significant relationship with unmet need, although the direction of effect implied that the less affluent participants tended to face greater odds of unmet need, compared with the well-off group. The original HWS questionnaire contains questions about household properties, and the surveyors were able to use the answers from these questions to estimate asset index. However, we dropped such questions in the questionnaire for URAS after we decided not to perform a physical visit to URAS households.

Fourthly, though the URAS survey and HWS followed the same set of questions, the timeline for conducting both surveys and human resources used were different. Therefore a direct comparison between URAS and Thais should take into account this limitation.

Fifthly, as per the intrinsic nature of cross-sectional design, it is difficult to identify causal relationship between unmet need and the selected independent variables. A cohort-based survey on URAS is recommended; but this requires the establishment of a system to regularly monitor health status of URAS over the long term. The system cannot be set up without collaboration amongst all concerned parties, especially the Thai government, NGOs, and the UNHCR. This raises a key issue mentioned earlier; whether the Thai government views URAS as a population it needs to take care of.

Sixthly, this study is not free from data bias, as some determinants between URAS and Thais were in stark contrast. The insurance variable was a clear example in this case. We found that less than 1% of the Thai participants were uninsured. In contrast, only 2.2% of URAS held some kind of health insurance. The lack of adequate case numbers for some combinations of exposure and outcome levels may cause an upward bias away from the null for the effect estimates [42]. This problem might also occur in other variables aside from insurance status, such as age groups and household prosperity. A more delicate analysis method, such as penalised regression, should be considered in further studies if the problem of sparse data appears again.

Lastly, the people of interest in this study were those presenting on the BRC roster only, not all URAS in Bangkok. We did not include URAS in non-household settings, such as shelters or detention centres. This definitely limits the generalisability power of our study. To expand the academic richness in this field, further studies on other types of refugees are strongly recommended.

## Conclusion

Overall, URAS had lower educational attainment and faced more severe financial hardship than Thais. The prevalence of unmet need in URAS was extremely high, relative to the corresponding prevalence in Thais. Factors that suggested a positive relationship with unmet need included advanced age, lower educational achievement, and, most evidently, being uninsured. All relevant parties, such as policy makers, academics and high-level bureaucrats in the public health area, should consider measures to include URAS in some kind of nationwide public insurance. The benefit of this it to alleviate unmet need for health services in URAS, but also to strengthen health security for Thai society as a whole. Additional studies on the health status and access to healthcare of other types of refugees are also recommended.

## Supplementary Information

**Additional file 1:** **Table S1.** Number of required samples and actual samples participating in the survey.

## Data Availability

The raw data used by this study jointly belonged to BRC and IHPP. The analysed data are however available from the authors upon reasonable request.

## Abbreviations

BRC: Bangkok Refugee Centre
CLM: Cambodia, Lao PDR and Myanmar
CI: Confidence interval
CSMBS: Civil Servant Medical Benefit Scheme
EU-SILC: European Union Statistics on Income and Living Conditions
HICS: Health Insurance Card Scheme
IHPP: International Health Policy Programme
IP: Inpatient
ME: Mixed-Effects
MOPH: Ministry of Public Health
NGO: Non-government Organisation
NV: Nationality Verification
OP: Outpatient
OR: Odds Ratio
OSS: One Stop Service
PPS: Probability Proportional to Size
SSS: Social Security Scheme
UHC: Universal Health Coverage
UNHCR: United Nations High Commissioner for Refugees
UN: United Nations
URAS: Urban Refugee and Asylum Seeker
WHA: World Health Assembly
WHO: World Health Organization
