# Supplementary material for: A cross sectional study of unmet need for health services amongst urban refugees and asylum seekers in Thailand in comparison with Thai population, 2019
Source: Int J Equity Health. 2020 Nov 11;19:205. doi: 10.1186/s12939-020-01316-y (PMC7661252; doi:10.1186/s12939-020-01316-y)
Supplement: Supplementary file 1 — Additional file 1: Table S1. Number of required samples and actual samples participating in the survey. [file 12939_2020_1316_MOESM1_ESM.pdf]

Supplementary table: Number of required samples and actual samples participating in the survey

National	Sex	Age (years)	Total number from BRC[#] list	Total samples required	Total participants attending the survey[*]
Afghan	Male	0-15	23	2	1
Afghan	Male	>15	26	2	3
Afghan	Female	0-15	16	2	1
Afghan	Female	>15	45	2	2
Cambodian	Male	0-15	30	2	2
Cambodian	Male	>15	72	4	4
Cambodian	Female	0-15	39	3	1
Cambodian	Female	>15	57	4	4
Chinese	Male	0-15	2	1	2
Chinese	Male	>15	22	2	1
Chinese	Female	0-15	14	2	1
Chinese	Female	>15	13	2	0
Iraqi	Male	0-15	12	2	0
Iraqi	Male	>15	32	2	2
Iraqi	Female	0-15	25	2	1
Iraqi	Female	>15	28	2	3
Sri Lankan	Male	0-15	13	2	2
Sri Lankan	Male	>15	30	2	0
Sri Lankan	Female	0-15	31	2	2
Sri Lankan	Female	>15	21	2	2
Pakistani	Male	0-15	243	15	14
Pakistani	Male	>15	392	25	22
Pakistani	Female	0-15	275	18	13
Pakistani	Female	>15	318	20	23
Palestinian	Male	0-15	37	2	1
Palestinian	Male	>15	56	4	0
Palestinian	Female	0-15	33	2	1
Palestinian	Female	>15	52	2	3
Somali	Male	0-15	40	2	2
Somali	Male	>15	72	5	1
Somali	Female	0-15	29	2	2
Somali	Female	>15	20	2	5
Vietnamese	Male	0-15	183	11	9
Vietnamese	Male	>15	250	16	13
Vietnamese	Female	0-15	197	13	11
Vietnamese	Female	>15	231	15	12
Syrian	Male	0-15	9	2	0
Syrian	Male	>15	12	2	3
Syrian	Female	0-15	11	2	2
Syrian	Female	>15	10	2	1

Note: In summary, we acquired 181 URASs but the rightmost comlum of the table displayed only 172 participants because nine URASs (one Chinese female, one Chinese male, one Palestinian male, three Vietnamese males and three Vietnamese females) did not have complete information on age and sex to specify in corresponding rows in the table. [#]BRC = Bangkok Refugee Center
